# Diagnostic accuracy of PLA2R autoantibodies and glomerular staining for the differentiation of idiopathic and secondary membranous nephropathy: an updated meta-analysis

**DOI:** 10.1038/srep08803

**Published:** 2015-03-05

**Authors:** Huanzi Dai, Huhai Zhang, Yani He

**Affiliations:** 1 Department of Nephrology, Daping Hospital, Third Military Medical University, Chongqing, PR China

## Abstract

The diagnostic performance of M-type phospholipase A2 receptor (PLA2R) autoantibodies and PLA2R glomerular staining in discriminating between idiopathic membranous nephropathy (iMN) and secondary membranous nephropathy (sMN) has not been fully evaluated. We conducted an updated meta-analysis to investigate the accuracy and clinical value of serological anti-PLA2R test and histological PLA2R staining for differentiation iMN from sMN. A total of 19 studies involving 1160 patients were included in this meta-analysis. The overall sensitivity, specificity, diagnostic odds ratio (DOR) and area under the receiver operating characteristic curve (AUROC) of serum anti-PLA2R were 0.68 (95% CI, 0.61–074), 0.97 (95% CI, 0.85–1.00), 73.75 (95% CI, 12.56–432.96) and 0.82 (95% CI, 0.78–0.85), respectively, with substantial heterogeneity (*I^2^* = 86.42%). Subgroup analyses revealed the study design, publication type, study origin, assay method might account for the heterogeneity. Additionally, the overall sensitivity, specificity, DOR and AUROC of glomerular PLA2R staining were 0.78 (95% CI, 0.72–0.83), 0.91 (95% CI, 0.75–0.97), 34.70 (95% CI, 9.93–121.30) and 0.84 (95% CI, 0.81–0.87), respectively, without heterogeneity (*I^2^* = 0%). Serological anti-PLA2R testing has diagnostic value, but it must be interpreted in context with patient clinical characteristics and histological PLA2R staining in seronegative patients is recommended.

Membranous nephropathy (MN), a common cause of adult nephrotic syndrome worldwide, can be idiopathic, or secondary to various clinical conditions, including systemic autoimmune disease, infections, neoplasia and drug intoxications^1^. Discriminating between these two groups of patients is of utmost clinical importance, since therapy in the sMN must be directed at the underlying cause and some of the treatments for iMN are potentially toxic both to the patient and the kidney^2^,^3^. To date, the diagnosis of iMN is still made by the exclusion of secondary causes using a detailed medical history, physical examination, laboratory studies and often invasive procedures^4^. However, in reality, differentiating iMN from sMN is difficult, especially in elderly patients in whom malignancies tend to occur^5^,^6^. Therefore, the need for an accurate biomarker to differentiate iMN from sMN is urgent.

In 2009, M-type phospholipase A2 receptor (PLA2R), a 185 kDa type I transmembrane glycoprotein expressed on glomerular podocytes, was identified as a major target antigen of the autoantibodies involved in iMN^7^. Circulating PLA2R autoantibodies were found in a majority (52–82%) of serum samples from patients with iMN, but absent in patients with sMN and other glomerular or autoimmune diseases, so these autoantibodies were suggested to not only play a direct pathogenic role but also be a promising marker for the differential diagnosis^8^,^9^,^10^,^11^,^12^,^13^,^14^. Furthermore, PLA2R staining were assessed in the renal biopsies and showed a good correlation with the serological test, although there was discordance in rare cases^12^,^13^,^15^,^16^.

However, with accumulating evidence, conflicting results have raised concerns about the clinical performance of serological anti-PLA2R and histological PLA2R staining tests for the diagnosis of iMN across various clinical situations. Thus, we performed a systematic review and meta-analysis to comprehensively investigate the diagnostic accuracy of the serological and histological tests to differentiate between iMN and sMN.

## Results

### Search results and study characteristics

**Figure 1:**
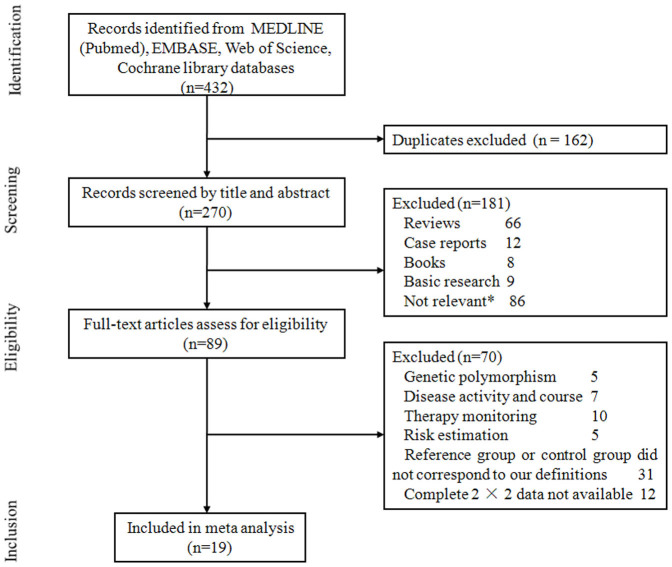
Flow chart of study selection. Some studies were excluded for more than one reason. *Did not investigate the diagnostic accuracy of PLA2R as a marker for iMN.

As shown in Figure 1, our search initially yielded 432 publications in total, with 162 duplicates. After screening titles and/or abstracts, another 181 articles were excluded, including reviews, case reports and basic research. 89 studies remained for evaluation via detailed reading. Among them, the topic of 27 studies did not focus on the diagnosis, and we could not extract data for a 2 × 2 quadrant table in 12 studies. The other 31 studies did not match inclusion criteria. Additional search of the reference lists of included studies and previous relevant reviews did not identify any articles. Finally, 19 studies were included in the analysis. 13^7^,^8^,^9^,^10^,^11^,^14^,^21^,^22^,^23^,^24^,^25^,^27^,^28^ of them only investigated the diagnostic value of anti-PLA2R detection, 3 studies^15^,^16^,^29^ only provided complete data for PLA2R glomerular deposits in the discernment between iMN and sMN, and 3 studies^12^,^13^,^26^ contained both serological and histological tests. Characteristics of included studies are listed in Table 1. A total of 1160 patients with MN were enrolled, and all the studies were conducted in adult patients.

### Quality assessment

**Figure 2:**
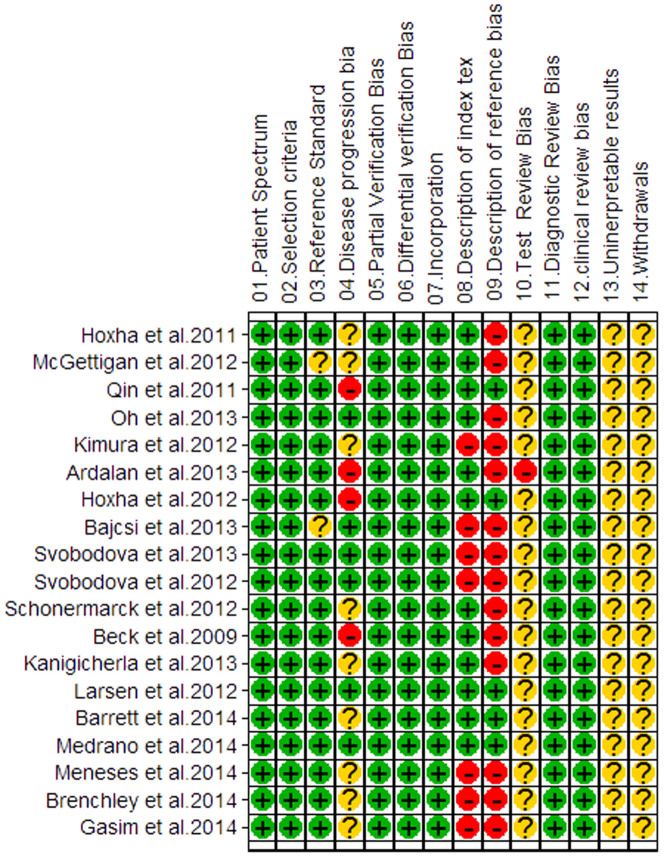
Methodological quality graph. Reviewer judgment of methodological quality of each individual study included in the analysis, according to the Quality Assessment of Diagnostic Accuracy Studies (QUADAS) tool.

The quality of the included studies according to the QUADAS standard is summarized in Figure 2. The inter-rater reliability for 14 items of QUADAS was 0.21 (p < 0.01). Overall, the methodological quality was moderate, with the scores ranging from 8 to 11. These studies were performed in Europe, America and Asia during 2009–2014, representing an international experience. 4^8^,^9^,^10^,^12^ of the studies were designed as prospective research, which possessed sufficient clinical information, whereas other studies were retrospective. 8 studies^8^,^14^,^21^,^22^,^25^,^26^,^27^,^28^ did not state the time of serum sampling relative to the detection by biopsy or whether immunosuppressors were administered prior to the serological tests. 10^7^,^8^,^9^,^10^,^11^,^12^,^13^,^14^,^24^,^25^,^26^ of the 19 studies reported classification of the secondary causes of MN, such as lupus, hepatitis B and malignant tumors. The levels of proteinuria and serum creatinine were reported in 9 studies^7^,^9^,^10^,^11^,^12^,^13^,^14^,^15^,^16^. 7 studies^8^,^11^,^12^,^13^,^21^,^24^,^25^ used an indirect immunofluorescence (IIF) assay to detect anti-PLA2R in serum, and only 4^7^,^9^,^10^,^23^ and 3^14^,^26^,^28^ studies used western blot (WB) and ELISA, respectively. However, most studies did not provide cutoff values.

### Data analysis and calculations

**Figure 3:**
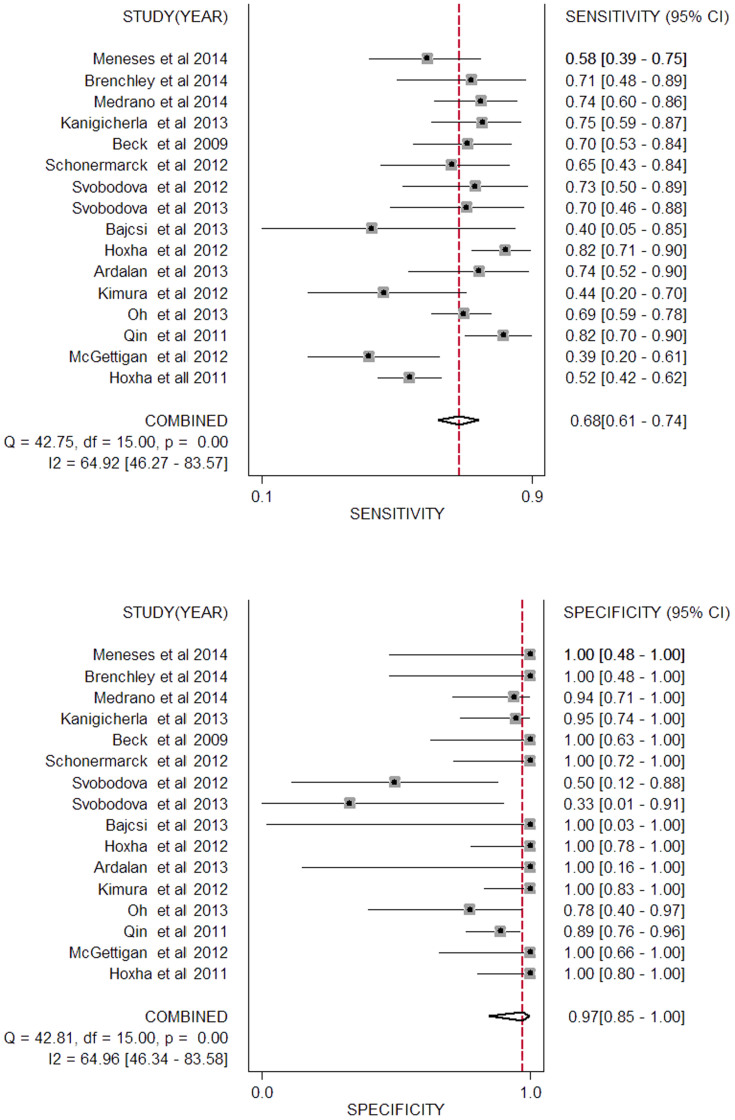
Forest plot of the pooled sensitivity and specificity of serum anti-PLA2R for differentiation iMN from sMN. The black squares in the gray squares and the horizontal lines represent the point estimate and 95% confidence interval (CI), respectively. The dotted line represents the pooled estimate, and the diamond shape represents the 95% CI of the pooled estimate.

**Figure 4:**
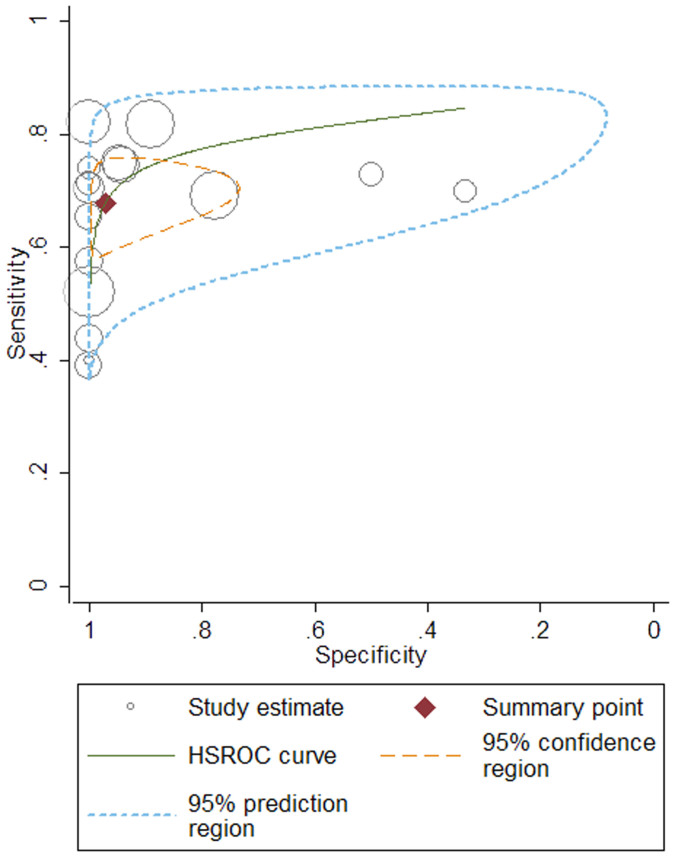
Hierarchical summary receiver operating characteristic (HSROC) plot of serum anti-PLA2R for differentiation iMN from sMN. The summary point represents the summary sensitivity and specificity, the 95% confidence region represents the 95% confidence intervals of the summary sensitivity and specificity and the 95% prediction region represents the 95% confidence interval of sensitivity and specificity of each individual study included in the analysis. The plot also includes study estimates indicating the sensitivity and specificity estimated using the data from each study separately. The size of the marker is scaled according to the total number in each study.

The true positive (TP), false positive (FP), true negative (TN), false negative (FN) and sensitivity and specificity of each study are listed in Table 2. Studies were stratified by different sample type, used to differentiate between iMN and sMN. As shown in Table 3, we found a DOR of 73.75 (95% CI, 12.56–432.96) for anti-PLA2R to differentiate iMN from sMN at a pooled sensitivity and specificity of 0.68 and 0.97 (Figure 3), respectively. The *I^2^* statistic was 83.70%, indicating significant heterogeneity across these studies. When patients were restricted to serum anti-PLA2R in conditions of >3.5 g/24 h proteinuria before immunesuppressor treatment at the time of renal biopsy (raw data shown in Table S1), the results revealed 0.78 for the sensitivity, 0.82 for the specificity, 16.54 for the DOR, 0.82 for AUC and *I^2^* statistic decreased to 0.00%. PLA2R staining in biopsy showed a DOR (34.70, 95% CI, 9.93–121.30), a sensitivity of 0.78 and a specificity of 0.91 without significant heterogeneity (*I^2^* = 0.00%). The area under the receiver operating characteristic curve (AUROC) was 0.82 (95% CI, 0.78–0.85; Figure 4) versus 0.84 (95% CI, 0.81–0.87) in serological and histological tests.

### Threshold effect and publication bias

The Spearman correlation coefficient of sensitivity and 1-specificity of the serological test was 0.16 (p = 0.55 > 0.05) suggesting that there is no threshold effect.

**Figure 5:**
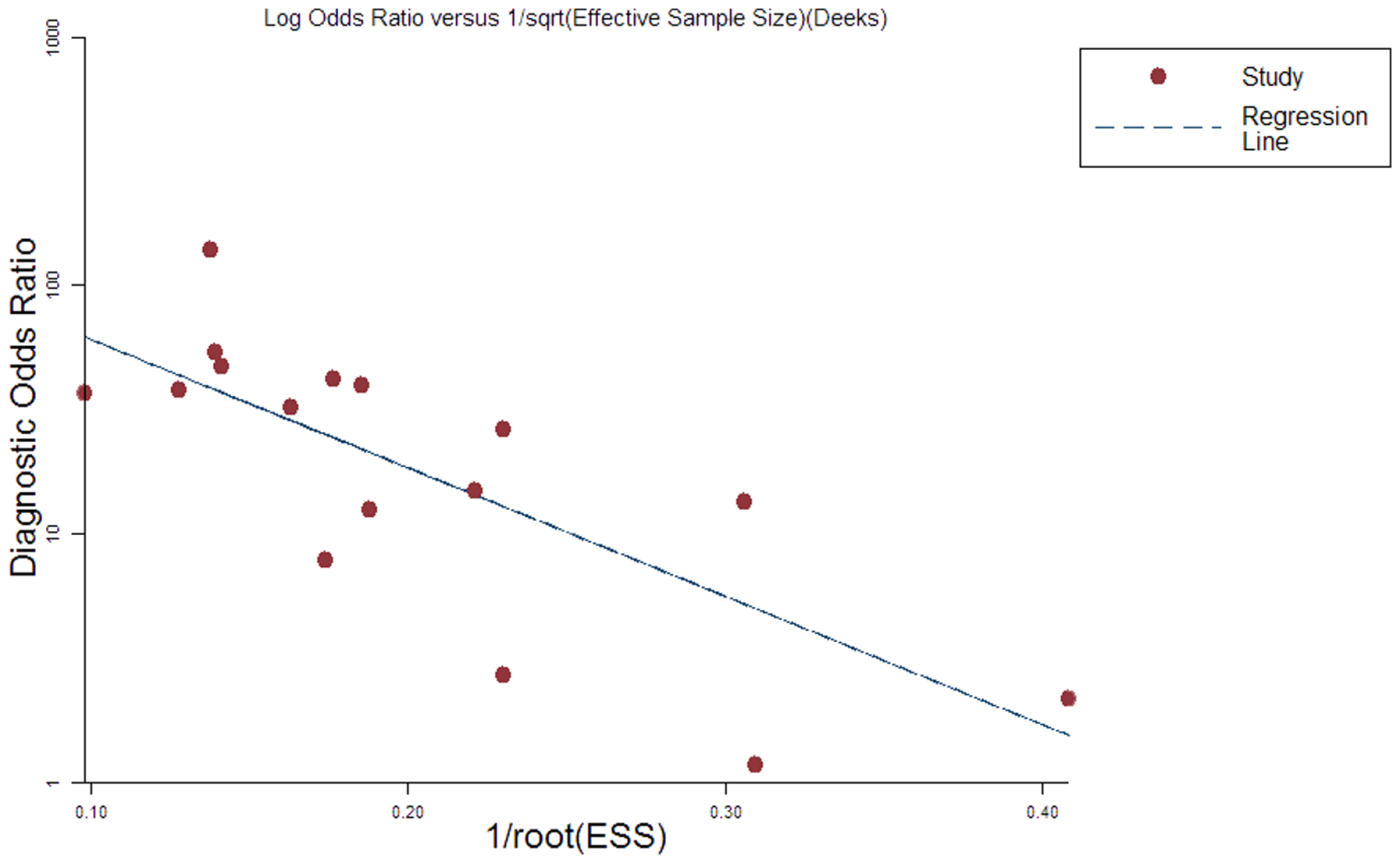
Deek's plots for included studies of serological anti-PLA2R test. Linear regression of log odds ratios on inverse root of effective sample sizes as a test for funnel plot asymmetry in diagnostic meta-analysis. A non-zero slope coefficient is suggestive of significant small study bias (p < 0.05).

As Deeks' funnel plot shown in Figure 5 (t = −3.41, *P* = 0.004), we observed the existence of asymmetry and a slope coefficient in funnel plots of these studies indicating that publication bias exists in the studies of serological tests. Several studies involved less than 100 patients which may be the main cause for the presence of publication bias.

### Subgroup analyses

Studies of serological tests were stratified into several subgroups (summary data shown in Table 4). The results revealed the study design, publication type, study origin, assay method might account for the heterogeneity.

### Serum anti-PLA2R and type of sMN

Three major causes of sMN involved in the included studies were SLE, hepatitis B and presence of a tumor (raw data shown in Table S2). The sensitivity, specificity, DOR and AUROC, respectively, were 0.70, 0.97, 65.19 and 0.97 when sMN was V type lupus MN, 0.74, 0.86, 17.58 and 0.83 when sMN was hepatitis B-related MN, 0.71, 0.81, 10.58 and 0.83 when sMN was tumor-associated MN.

### Serum anti-PLA2R and proteinuria levels

We divided the patients into nephrotic syndrome group and non-nephrotic syndrome group according to proteinuria levels (raw data shown in Table S3). The sensitivity, specificity and DOR, respectively, were 0.77, 0.91 and 34.44 for patients with nephrotic syndrome and 0.32, 0.91 and 4.77 for patients with non-nephrotic syndrome. The AUROC was much higher in the patients with nephrotic syndrome (0.83 vs 0.47).

### Serum anti-PLA2R and immunosuppressant therapy

Considering affections of immunosuppressant therapy (data shown in Table S4), we compared the sensitivity, specificity and DOR in the patients who were treated with and without immunosuppressant therapies (0.44, 0.93 and 10.79 versus 0.72, 0.89 and 20.21, respectively).

### Serum anti-PLA2R and sampling time from biopsy

The sensitivity, specificity and DOR, respectively, were 0.73, 0.87 and 17.99 for the patients when the sampling time was at the day of biopsy and 0.52, 0.95 and 19.37 for the patients when the sampling time was several times after the day of the biopsy (data shown in Table S5).

## Discussion

Serum anti-PLA2R level or glomerular PLA2R staining diagnostic accuracy in iMN was investigated previously, with heavily biased results^26^,^27^. One meta-analyses^26^ enrolled healthy individuals and patients without MN (non-kidney diseases or other glomerular diseases) as controls, possibly increasing the overall diagnostic accuracy estimate. We included only patients with sMN as controls, directly assessing whether serum anti-PLA2R and histological PLA2R staining can discriminate between iMN and sMN correctly. The quantitative data analysis by Hu and colleagues^26^ summarized sensitivity and specificity into one diagnostic accuracy measure. To retain the two-dimensional character, we used the optimal statistical methods of combining the studies, i.e., bivariate mixed effects regression models and HSROC. Moreover, they included only three studies for predicting active-stage iMN, two studies for biopsy testing, and two datasets from the same patient group to combine effects, therefore their conclusion should be considered with caution. They also said little about the accuracy of high-heterogeneity summary estimates, which we investigated using subgroup analyses. Du and colleagues^27^ also used a healthy population and patients with other kidney diseases as controls. Some data extracted for meta-analyses differed from the inclusion criteria: they excluded patients with non-kidney diseases or who received immunosuppressive therapy, but such patients were included from the studies of Beck^7^ and Hoxha^8^. Additionally, their conclusion was conflicted that the possible confounders therapeutic intervention and disease progression during the testing interval may have contributed to the heterogeneity. Furthermore, study of Dähnrich and colleagues^28^ should not be used to calculate sensitivity data, as their “iMN” patients were pre-selected to be anti-PLA2R-positive, leading to overestimation in the given calculation. Thus, these mentioned above greatly affected their overall conclusions.

Overall, serological anti-PLA2R testing had diagnostic value in discriminating iMN from sMN. According to proteinuria subgroup analysis, anti-PLA2R testing had much greater diagnostic accuracy for nephrotic syndrome (AUC = 0.83) than for non-nephrotic syndrome (AUC = 0.47). Anti-PLA2R levels may fluctuate with disease activity^14^,^29^,^30^; serum anti-PLA2R decreases spontaneously and even faster under immunosuppressive therapy (the positive rate decreases to 15.79% ~ 28.57%) (Table S6). Then, primary and secondary MN cannot be distinguished when anti-PLA2R becomes negative. Subgroup analyses also showed that the pooled specificity was low, only 0.44 and 0.52 for patients treated and sampled after biopsy respectively, indicating immunosuppressive therapy could have removed the antibodies or spontaneous remission with the consequent antibody disappearance could have occurred after a long time interval following renal biopsy. Our subsequent subgroup analyses included only patients with nephrotic-range proteinuria before immunosuppressive therapy at renal biopsy, a more homogeneous group (*I*^2^ = 0.00%): serum anti-PLA2R testing performed well (AUC = 0.82) (Table 3). However, including these patients may much accord with actual clinical situations; such investigations regarding serum anti-PLA2R diagnostic value should be interpreted with caution.

The possible explanations of biopsy testing is more sensitive than serological testing for diagnosing iMN are rapid antibodies clearance from the blood and deposition in the glomeruli, or late sampling when proteinuria persisted because of irreversible ultrastructural changes^8^,^31^,^32^,^33^. Therefore, extended screening for PLA2R staining in the glomeruli is recommended in seronegative patients.

The data were significantly discrepant between different forms of sMN. AUC = 0.97 represented the highest diagnostic accuracy when sMN was lupus MN. Moreover, the form of sMN significantly influenced the FP rate, although iMN coinciding with the associated disease cannot be excluded. Anti-PLA2R FNs would not have resulted in severe outcomes in all patients, as spontaneous remission occurs in 30–40% of patients, but anti-PLA2R FPs could result in harmful treatments and delayed/no detection of primary diseases, especially underlying malignancy in older patients with MN^34^. Hence, routine age- and sex-appropriate malignancy screening is necessary for older patients with newly diagnosed MN, even serological anti-PLA2R- and/or histological PLA2R antigen-positive patients.

In our study, positive LRs of 24.48 and 8.40 implied that a person with iMN was 24.48 (serological testing) and 8.40 (biopsy testing) times likelier, respectively, to have a positive result than a person with sMN. Given a 25% pretest probability, the post-test probability of a positive test result was 89% (serological testing) and 74% (biopsy testing). Meanwhile, negative LRs of 0.33 and 0.24 reduced the post-test probability of a negative result to 10% (serological testing) and 7% (biopsy testing) (Table 3). However, these LRs were calculated from dichotomized data: the result is either positive or negative. The disadvantage is that useful information is lost. To obtain more precise information, we suggest calculating LRs using multiple cutoffs.

Our study has several limitations. Firstly, the methodological quality of included studies was moderate: many had potential verification or disease progression biases. In some studies, implementation was poorly reported, especially test review bias, uninterpretable results and withdrawals. As there is currently no common validated measurement platform, inter-study assay methods varied. Furthermore, WB and IIF signal intensity are difficult to standardize because there are no guidelines/criteria for establishing a diagnostic cutoff value. Future diagnostic accuracy studies require ELISA standardization, which defines the normal range and objective threshold for discriminating positive and negative results in clinical studies.

Secondly, the considerable amount of heterogeneity was detected among the studies of serological tests. Our subgroup analyses found that retrospective study design (*I^2^* = 79.81%, *P* = 0.004), abstract publication (*I^2^* = 79.59%, *P* = 0.004), small sample size (*I^2^* = 81.50%, *P* = 0.002), European/American study (*I^2^* = 80.80%, *P* = 0.003) and IIF (*I^2^* = 84.24%, *P* = 0.001) were responsible for the heterogeneity. Additionally, unrecorded inter-study differences probably contributed to it. Including a more homogenous population would resolve this, but would cause selection bias.

Thirdly, only six histological testing studies were included, and the available information was insufficient for subgroup analyses, therefore it was difficult to draw a definitive conclusion for its ability to discriminate. Hence, biopsy testing requires further study. Additionally, we only included English-language articles, thus language bias may have influenced the results.

In conclusion, this meta-analysis suggests that serological anti-PLA2R testing has diagnostic value for differentiating iMN from sMN, but it must be interpreted in context with patient clinical characteristics (degree of proteinuria, immunosuppressive treatment, time of detection). Histological PLA2R staining in seronegative patients is recommended. Studies included were nonrandomized, and potential confounders cannot be strictly controlled. Thus, well-designed prospective studies with large patients cohorts are required to reliably evaluate the value of anti-PLA2R and PLA2R antigen for identifying iMN.

## Methods

This systematic review and meta-analysis was performed in accordance with the Preferred Reporting Items for Systematic reviews and Meta-Analyses (PRISMA) guidelines^35^.

### Data sources and searches

We searched MEDLINE (PubMed), EMBASE, Web of Science and Cochrane library databases until October 2014 using following search terms with English language: PLA2R (phospholipase A2 receptor or M-type phospholipase A2 receptor) and MN (membranous nephropathy, membranous glomerulonephritis or membranous glomerulopathy). Manual searches were conducted following reviews of the reference lists of all selected articles to identify any missing studies.

### Study selection, data extraction, quality assessment and data synthesis

Studies were included if they assessed anti-PLA2R in serum and/or PLA2R in glomeruli for differentiation between iMN and sMN. To be eligible, studies had to have a well defined reference standard, which included patients were designated as sMN if they had confirmed etiologies of MN and designated as idiopathic after exclusion of known secondary etiologies through history, physical exam, and laboratory tests and kidney biopsy (including light, electron microscopy and immunofluorescence). Morever, the studies had to provide sufficient information for the 2 × 2 contingency table. Conference abstracts could be included if they contained available data. Study selection, data extraction, quality assessment, and data synthesis were independently performed by two reviewers, Dai and Zhang, Any disagreements were resolved through discussion or a third reviewer, He. Characteristics of included studies and data of 2 × 2 contingency tables were extracted. The Quality Assessment of Studies of Diagnostic Accuracy included in Systematic Review (QUADAS) assessment tool^36^, which contains 14 items was applied for the quality assessment of the included studies. A bivariate mixed-effects model of meta-analytical integration of diagnostic accuracy studies (MIDAS) module in STATA (version 12.0) was used for calculation of sensitivity, specificity, diagnostic odds ratio (DOR), area under the receiver operating characteristic curve (AUROC) and positive and negative likelihood ratios (LRs). A hierarchical summary receiver operating curve (HSROC) was constructed with the derived logit estimates of sensitivity, specificity^37^. The post-test probability with assigned pre-test probability of 25% was calculated based on the pooled sensitivity and specificity. The κ statistic was calculated for the inter-rater reliability between two investigators for quality assessment. The *I^2^* was used to assess heterogeneity with *I^2^* > 50% indicating the presence of significant heterogeneity. For Q test, P value less than 0.05 stands for significant heterogeneity. To explore the potential source of heterogeneity, we stratified the studies into several subgroups (according to characteristics of studies and patients, including type of publication, country of origin, study design, sample size, proteinuria, treatment with or without immunosuppressor, type of sMN, interval between biopsy and serum sample) and calculated specificity, sensitivity and relevant parameters. Publication bias was investigated by Deek's plot and considered to be present if there was a non-zero slope coefficient (*P* < 0.05)^38^.

## Supplementary Material

Supplementary Information Supplementary Information

## Figures and Tables

**Table 1:** Characteristics of included studies

Study, Year(Reference)	Research type	Publication	Area	Age^a^	Sex (male/female)	Samplesize	Sample type	Proteinuria (g/day)^a^^b^	Serum Creatinine (mg/dl)^a^^b^	TreatmentWith IS^a^	Time from biopsy(Mo.)
Hoxha et al. 2011^8^	Prospective	Full txt	Germany	NA	NA	117	Serum	“nonNS-NS”	NA	55%&29%	NA
McGettigan et al. 2012^17^	Retrospective	Abstract	Australia	NA	NA	32	Serum	NA	NA	NA	NA
Qin et al. 2011^9^	Prospective	Full txt	China	41	61/35^c^	106	Serum	3.51–13.5&3.51–14.43	0.4–3.92&0.22–1.46	None	0
Oh et al. 2013^10^	Prospective	Full txt	Korea	54.7	51/49^c^	109	Serum	3.17–9.86	0.91 ± 0.35	None	0
Kimura et al. 2012^18^	Retrospective	Abstract	Japan	NA	NA	36	Serum	NA	NA	NA	NA
Ardalan et al. 2013^11^	Retrospective	Full txt	Iran	34	13/12	25	Serum	0.7–4.9	1.0–9.5	all	7–71
Hoxha et al. 2012^12^	Prospective	Full txt	Germany	56.5&57.7	64/24	88	Serum + Tissue	0.1–33&0.77–22.7	0.5–5.28&0.7–2.7	None	0–11
Bajcsi et al. 2013^19^	Retrospective	Abstract	Hungary	NA	NA	6	Serum	NA	NA	NA	0
Svobodova et al. 2013^13^	Retrospective	Full txt	Czech	52.6	NA	84	Serum + Tissue	10.1 ± 7.6	1.22 ± 0.58	None	0
Svobodova et al. 2012^20^	Retrospective	Abstract	Czech	NA	NA	28	Serum	8.6 ± 3.6&9.3 ± 8.3	NA	None	0
Schonermarck et al. 2012^21^	Retrospective	Abstract	Germany	NA	NA	34	Serum	NA	NA	NA	NA
Beck et al. 2009^7^	Retrospective	Full txt	USA	47&38	28/17	45	Serum	0.6–14&0.3–9.1	0.4–3.1&0.5–0.8	34%&43%	0–132
Kanigicherla et al. 2013^14^	Retrospective	Full txt	UK	54	64/26^c^	109	Serum	5.9–12.8	0.86–1.37	30%	NA
Larsen et al. 2012^16^	Retrospective	Full txt	USA	57.5&47.4	89/76	165	Tissue	7.3 ± 5&5.7 ± 4.7	1.4 ± 0.9 &1.7 ± 1.7	NA	0
Barrett et al. 2014^15^	Retrospective	Full txt	USA	69	3/4	7	Tissue	1–11&1.5–9	1.2–4.1&1.1–5.7	all	0
Medrano et al. 2014^22^	Retrospective	Full txt	Spain	52.3	41/23	64	Serum + Tissue	8.6–15&7.55–11	1.1 ± 0.55&1 ± 0.27	NA	NA
Meneses et al. 2014^23^	Retrospective	Abstract	Portugal	NA	NA	38	Serum	NA	NA	NA	NA
Brenchley et al. 2014^24^	Retrospective	Abstract	UK	NA	NA	26	Serum	NA	NA	NA	NA
Gasim et al. 2014^25^	Retrospective	Abstract	USA	54	21/20	41	Tissue	NA	NA	NA	NA

Abbreviations: NS, nephrotic syndrome; IS, immunosuppressor; NA, not available; Mo, month.

^a^A ampersand separating values denotes data from idiopathic membranous nephropathy and secondary membranous nephropathy respectively.

^b^data were expressed as an statistic range or mean ± standard deviation.

^c^data were only provided from patients with idiopathic membranous nephropathy.

**Table 2:** Sensitivity and specificity of anti-PLA2R and PLA2R staining for differentiation iMN from sMN

Study(Author. Year)	Cut off value	TP	FP	FN	TN	Assay method	Sensitivity	Specificity
**Serological tests**								
Hoxha et al. 2011^8^	N.A	52	0	48	17	IIF	0.52	1.00
McGettigan et al. 2012^17^	N.A	9	0	14	9	IIF	0.39	1.00
Qin et al. 2011^9^	N.A	49	5	11	41	WB	0.82	0.89
Oh et al. 2013^10^	N.A	69	2	31	7	WB	0.69	0.78
Kimura et al. 2012^18^	N.A	7	0	9	20	WB	0.44	1.00
Ardalan et al. 2013^11^	N.A	17	0	6	2	IIF	0.74	1.00
Hoxha et al. 2012^12^	N.A	60	0	13	15	IIF	0.82	1.00
Bajcsi et al. 2013^19^	N.A	2	0	3	1	N.A	0.40	1.00
Svobodova et al. 2013^13^	N.A	14	2	6	1	IIF	0.70	0.33
Svobodova et al. 2012^20^	N.A	16	3	6	3	IIF	0.73	0.50
Schonermarck et al. 2012^21^	N.A	15	0	8	11	IIF	0.65	1.00
Beck et al. 2009^7^	N.A	26	0	11	8	WB	0.70	1.00
Kanigicherla et al. 2013^14^	N.A	30	1	10	18	ELISA	0.75	0.95
Medrano et al. 2014^22^	15 RU/ml	35	1	12	16	ELISA	0.74	0.94
Brenchley et al. 2014^24^	N.A	15	0	6	5	ELISA	0.71	1.00
Meneses et al. 2014^23^	N.A	19	0	14	5	N.A	0.58	1.00
**Biopsy tests**								
Hoxha et al. 2012^12^	N.A	61	0	12	15	IHC	0.84	1.00
Svobodova et al. 2013^13^	N.A	16	3	4	16	IIF	0.80	0.84
Larsen et al. 2012^16^	N.A	64	14	21	66	IIF	0.75	0.83
Barrett et al. 2014^15^	N.A	2	0	1	4	IIF	0.67	1.00
Gasim et al. 2014^25^	N.A	25	0	9	7	IIF	0.74	1.00
Medrano et al. 2014^22^	N.A	36	1	11	16	IHC	0.77	0.94

Abbreviations: TP, true positive; FP, false positive; TN, true negative. FN, false negative; PPV, positive predictive value; NPV, negative predictive value; WB, western blot; IIF, indirect immunofluorescence; IHC, immunohistochemistry; ELISA, enzyme linked immunosorbent assay; NA, not available.

**Table 3:** Pooled diagnostic accuracy for differentiation iMN from sMN

PLA2R	No. of studies	Pooled Sensitivity (95%CI)	Pooled Specificity (95%CI)	DOR (95% CI)	AUROC (95% CI)	*I^2^* (%)	Likelihood Ratio (95% CI)		Pre-test	Post-test(+)	Post-test(−)
							Positive	Negative			
Serological tests^a^	16	0.68(0.61–0.74)	0.97(0.85–1.00)	73.75(12.56–432.96)	0.82(0.78–0.85)	83.70	24.48(4.21–142.43)	0.33(0.28–0.40)	0.25	0.89	0.10
Serological tests^b^	5	0.78(0.71–0.84)	0.82(0.61–0.93)	16.54(4.82–56.78)	0.82(0.78–0.85)	0.00	4.38(1.74–10.79)	0.26(0.18–0.39)	0.25	0.59	0.08
Biopsy tests	6	0.78(0.72–0.83)	0.91(0.75–0.97)	34.70(9.93–121.30)	0.84(0.81–0.87)	0.00	8.40(2.87–24.64)	0.24(0.18–0.32)	0.25	0.74	0.07

^a^: data from serum anti-PLA2R in patients of included articles.

^b^: data from serum anti-PLA2R in patients of >3.5 g/24 h proteinuria before immunosuppressor treatment at the time of renal biopsy.

Abbreviations: *I^2^*, chi-square; CI, confidence interval; DOR, diagnostic odds ratio; AUROC, area under the receiver operating characteristic curve.

**Table 4:** Diagnostic accuracy of serum anti-PLA2R in various analyzed subgroups

Subgroup	No. of studies	Pooled Sensitivity (95%CI)	Pooled Specificity (95%CI)	DOR (95% CI)	AUROC (95% CI)	P value	*I^2^* (%)	Likelihood Ratio (95% CI)		Pre-test	Post-test(+)	Post-test(−)
								Positive	Negative			
**Study design**												
**Prospective**	4	0.72(0.59–0.82)	0.93(0.83–0.97)	33. 89(12.43–92.34)	0.93(0.91–0.95)	0.089	42.16	10.20(4.15–25.09)	0.30(0.20–0.46)	0.25	0.77	0.09
**Retrospective**	12	0.66(0.59–0.73)	0.99(0.76–1.00)	127. 49(7.05–2303.89)	0.77(0.73–0.80)	0.004	79.81	44.09(2.39–814.62)	0.35(0.28–0.42)	0.25	0.94	0.10
**Publication**												
**Full text**	9	0.72(0.65–0.79)	0.93(0.86–0.96)	32. 22(15.34–67.70)	0.93(0.90–0.95)	0.141	20.95	9.62(4.97–18.62)	0.30(0.24–0.38)	0.25	0.76	0.09
**Abstract**	7	0.58(0.47–0.68)	1.00(0.16–1.00)	1034. 68(0.30–3.6e + 06)	0.71(0.67–0.75)	0.004	79.57	436.74(0.12–1.6e + 06)	0.42(0.33–0.54)	0.25	0.99	0.12
**Sample size**												
**≥100**	4	0.69(0.57–0.79)	0.93(0.82–0.97)	29.44(11.06–78.35)	0.91(0.88–0.93)	0.053	55.42	9.72(3.86–24.49)	0.33(0.23–0.47)	0.25	0.76	0.10
**<100**	12	0.67(0.57–0.74)	0.99(0.69–1.00)	260.07(4.77–1.4e + 04)	0.80(0.76–0.83)	0.002	81.50	86.71(1.59–4717.38)	0.33(0.26–0.42)	0.25	0.97	0.10
**Area of origin**												
Europe and **America**	13	0.67(0.60–0.74)	0.99(0.82–1.00)	201.40(9.52–4261.19)	0.80(0.77–0.84)	0.003	80.80	66.57(3.15–1408.79)	0.33(0.27–0.41)	0.25	0.96	0.10
**Asia**	4	0.69(0.56–0.80)	0.93(0.80–0.98)	29.07(9.95–84.93)	0.90(0.88–0.93)	0.169	7.61	9.61(3.04–26.90)	0.33(0.23–0.48)	0.25	0.76	0.10
**Assay methods**												
**WB**	4	0.69(0.56–0.79)	0.94(0.81–0.98)	32.87(10.30–104.93)	0.90(0.87–0.92)	0.168	8.39	10.98(3.52–34.26)	0.33(0.24–0.48)	0.25	0.79	0.10
**IIF**	7	0.67(0.55–0.77)	0.99(0.27–1.00)	223.84(0.79–6.3e + 04)	0.79(0.75–0.82)	0.001	84.24	75.48(0.27–2.1e + 04)	0.34(0.24–0.47)	0.25	0.96	0.10
**Secondary forms of MN**												
**Lupus V**	8	0.70(0.59–0.77)	0.97(0.87–0.99)	65.19(14.28–297.54)	0.97(0.95–0.98)	0.385	0.00	20.94(5.13–85.46)	0.32(0.24–0.44)	0.25	0.87	0.10
**Hepatitis B**	8	0.74(0.69–0.79)	0.86(0.66–0.95)	17.58(5.09–60.71)	0.83(0.79–0.86)	0.447	0.00	5.27(1.91–14.55)	0.30(0.23–0.40)	0.25	0.64	0.09
**Tumor**	6	0.71(0.61–0.80)	0.81(0.51–0.95)	10.58(2.50–44.95)	0.83(0.79–0.86)	0.308	0.00	3.75(1.21–11.64)	0.36(0.24–0.53)	0.25	0.56	0.11
**Level of proteinuria**												
**≥3.5 g/d**	10	0.77(0.72–0.82)	0.91(0.76–0.97)	34.44(9.56–124.08)	0.83(0.79–0.86)	0.123	28.81	8.62(2.95–25.19)	0.25(0.19–0.33)	0.25	0.74	0.08
**<3.5 g/d**	9	0.32(0.23–0.43)	0.91(0.51–0.99)	4.77(0.37–61.99)	0.47(0.43–0.52)	0.198	0.00	3.57(0.37–34.61)	0.75(0.55–1.02)	0.25	0.54	0.20
**Treatment with IS before anti-PLA2R assay**												
**Untreat**	7	0.72(0.63–0.80)	0.89(0.62–0.97)	20.21(4.17–98.09)	0.81(0.78–0.84)	0.097	39.17	6.31(1.62–24.67)	0.31(0.22–0.44)	0.25	0.68	0.09
**Treat**	7	0.44(0.72–0.98)	0.93(0.72–0.99)	10.79(1.36–85.65)	0.80(0.76–0.83)	0.486	0.00	6.46 (1.124–37.42)	0.60(0.41–0.88)	0.25	0.68	0.17
**Sampling time from biopsy (mo)**												
**<1**	5	0.73(0.66–0.80)	0.87(0.73–0.94)	17.99(6.83–47.36)	0.87(0.84–0.90)	0.498	0.00	5.57(2.56–12.11)	0.31(0.23–0.41)	0.25	0.65	0.09
**≥1**	9	0.52(0.33–0.70)	0.95(0.67–0.99)	19.37(1.35–278.99)	0.82(0.78–0.85)	0.364	0.00	9.86(1.01–96.44)	0.51(0.32–0.81)	0.25	0.71	0.11

Abbreviations: CI, confidence interval; DOR, diagnostic odds ratio; AUROC, area under the receiver operating characteristic curve; *I^2^*, chi-square; WB, western blot;

IF, immunofluorescence; MN, membranous nephropathy; IS, immunosuppressor; Mo, month.
