# Supplementary material for: Diagnostic accuracy of PLA2R autoantibodies and glomerular staining for the differentiation of idiopathic and secondary membranous nephropathy: an updated meta-analysis
Source: Sci Rep. 2015 Mar 5;5:8803. doi: 10.1038/srep08803 (PMC4350087; doi:10.1038/srep08803)
Supplement: Supplementary Information [file srep08803-s1.docx]

Diagnostic accuracy of PLA2R autoantibodies and glomerular staining for the differentiation of idiopathic and secondary membranous nephropathy: an updated meta-analysis

**Huanzi Dai* , Huhai Zhang* & Yani He**

*Note: Huanzi Dai and Huhai Zhang contributed equally to this study

**Table S1. Raw data from serum anti-PLA2R in patients of >3.5g/24h proteinuria before immunosuppressor treatment at the time of renal biopsy**

|  | **Number of patient** | | | | **Sensitivity (95%CI)** | **Specificity (95%CI)** |
| --- | --- | --- | --- | --- | --- | --- |
|  | **TP** | **FP** | **FN** | **TN** |  |  |
| Qin et al.2011 ^9^ | 49 | 4 | 11 | 39 | 0.82(0.70-0.90) | 0.91(0.78-0.97) |
| Oh et al.2013 ^10^ | 60 | 2 | 15 | 7 | 0.80(0.69-0.88) | 0.78(0.40-0.97) |
| Hoxha et al.2012 ^12^ | 17 | 0 | 4 | 7 | 0.81(0.58-0.95) | 1.00(0.59-1.00) |
| Svobodova et al.2013 ^13^ | 14 | 1 | 6 | 1 | 0.70(0.46-0.88) | 0.50(0.01-0.99) |
| Svobodova et al.2012 ^20^ | 16 | 3 | 6 | 3 | 0.73(0.50-0.89) | 0.50(0.12-0.88) |

Abbreviations: TP, true positive; FP, false positive; TN, true negative. FN, false negative; CI, confidence interval.

**Table S2.Raw data from serum anti-PLA2R in subgroups of different causes of sMN**

| **Secondary MN** | **Number of patient** | | | | **Sensitivity (95%CI)** | **Specificity (95%CI)** |
| --- | --- | --- | --- | --- | --- | --- |
|  | **TP** | **FP** | **FN** | **TN** |  |  |
| **Lupus type V** |  |  |  |  |  |  |
| Hoxha et al.2011 ^8^ | 52 | 0 | 48 | 6 | 0.52(0.42-0.62) | 1.00(0.54-1.00) |
| Qin et al.2011 ^9^ | 49 | 1 | 11 | 19 | 0.82(0.70-1.00) | 0.95(0.75-1.00) |
| Oh et al.2013 ^10^ | 69 | 0 | 31 | 1 | 0.69(0.59-0.78) | 1.00(0.03-1.00) |
| Hoxha et al.2012 ^12^ | 60 | 0 | 13 | 5 | 0.82(0.71-0.90) | 1.00(0.48-1.00) |
| Svobodova et al.2012 ^20^ | 16 | 0 | 6 | 1 | 0.73(0.50-0.89) | 1.00(0.03-1.00) |
| Beck et al.2009 ^7^ | 26 | 0 | 11 | 6 | 0.70(0.53-0.84) | 1.00(0.54-1.00) |
| Kanigicherla et al.2013 ^14^ | 42 | 1 | 48 | 18 | 0.47(0.36-0.57) | 0.95(0.74-1.00) |
| Ardalan et al.2013 ^11^ | 17 | 0 | 6 | 1 | 0.74(0.52-0.90) | 1.00(0.03-1.00) |
| **Hepatitis B** |  |  |  |  |  |  |
| Qin et al.2011 ^9^ | 49 | 1 | 11 | 15 | 0.82(0.70-0.90) | 0.94(0.70-1.00) |
| Oh et al.2013 ^10^ | 69 | 1 | 31 | 4 | 0.69(0.59-0.78) | 0.80(0.28-0.99) |
| Hoxha et al.2012 ^12^ | 60 | 0 | 13 | 1 | 0.82(0.71-0.90) | 1.00(0.03-1.00) |
| Svobodova et al.2013 ^13^ | 14 | 1 | 6 | 1 | 0.70(0.46-0.88) | 0.50(0.01-0.99) |
| Svobodova et al.2012 ^20^ | 16 | 1 | 6 | 1 | 0.73(0.50-0.89) | 0.50(0.01-0.99) |
| Schonermarck et al.2012 ^21^ | 15 | 0 | 8 | 1 | 0.65(0.43-0.84) | 1.00(0.03-1.00) |
| Beck et al.2009 ^7^ | 26 | 0 | 11 | 2 | 0.70(0.53-0.84) | 1.00(0.16-1.00) |
| Ardalan et al.2013 ^11^ | 17 | 0 | 6 | 1 | 0.74(0.52-0.90) | 1.00(0.03-1.00) |
| **Tumor** |  |  |  |  |  |  |
| Hoxha et al.2011 ^8^ | 52 | 0 | 48 | 3 | 0.52(0.42-0.62) | 1.00(0.29-1.00) |
| Qin et al.2011 ^9^ | 49 | 3 | 11 | 7 | 0.82(0.70-0.90) | 0.70(0.35-0.93) |
| Oh et al.2013 ^10^ | 69 | 1 | 31 | 1 | 0.69(0.59-0.78) | 0.50(0.01-0.99) |
| Hoxha et al.2012 ^12^ | 60 | 0 | 13 | 7 | 0.82(0.71-0.90) | 1.00(0.59-1.00) |
| Svobodova et al.2012 ^20^ | 16 | 1 | 6 | 0 | 0.73(0.50-0.89) | 0.00(0.00-0.98) |
| Schonermarck et al.2012 ^21^ | 15 | 0 | 8 | 1 | 0.65(0.43-0.84) | 1.00(0.03-1.00) |

Abbreviations: MN, membranous nephropathy; TP, true positive; FP, false positive; TN, true negative. FN, false negative; CI,

confidence interval.

**Table S3.Raw data from serum anti-PLA2R in subgroups of different proteinuria levels**

| **Proteinuria** | **Number of patient** | | | | **Sensitivity (95%CI)** | **Specificity (95%CI)** |
| --- | --- | --- | --- | --- | --- | --- |
|  | **TP** | **FP** | **FN** | **TN** |  |  |
| **≥3.5g/d** |  |  |  |  |  |  |
| Hoxha et al.2011 ^8^ | 23 | 0 | 12 | 12 | 0.66(0.48-0.81) | 1.00(0.74-1.00) |
| Qin et al.2011 ^9^ | 49 | 4 | 11 | 39 | 0.82(0.70-0.90) | 0.91(0.78-0.97) |
| Oh et al.2013 ^10^ | 60 | 2 | 15 | 7 | 0.80(0.69-0.88) | 0.78(0.40-0.97) |
| Hoxha et al.2012 ^12^ | 55 | 0 | 10 | 13 | 0.85(0.74-0.92) | 1.00(0.85-1.00) |
| Svobodova et al.2013 ^13^ | 18 | 2 | 10 | 1 | 0.64(0.44-0.81) | 0.33(0.01-0.91) |
| Svobodova et al.2012 ^20^ | 22 | 3 | 10 | 3 | 0.69(0.50-0.84) | 0.50(0.12-0.88) |
| Beck et al.2009 ^7^ | 17 | 0 | 3 | 4 | 0.85(0.62-0.97) | 1.00(0.40-1.00) |
| Kanigicherla et al.2013 ^14^ | 30 | 1 | 10 | 18 | 0.75(0.59-0.87) | 0.95(0.74-1.00) |
| Ardalan et al.2013 ^11^ | 6 | 0 | 0 | 2 | 1.00(0.54-1.00) | 1.00(0.16-1.00) |
| Medrano et al.2014^22^ | 35 | 1 | 12 | 16 | 0.74(0.60-0.86) | 0.94(0.71-1.00) |
| **＜3.5g/d** |  |  |  |  |  |  |
| Hoxha et al.2011 ^8^ | 3 | 0 | 14 | 5 | 0.18(0.04-0.43) | 1.00(0.48-1.00) |
| Qin et al.2011 ^9^ | 4 | 1 | 17 | 2 | 0.19(0.05-0.42) | 0.67(0.09-0.99) |
| Oh et al.2013 ^10^ | 12 | 2 | 32 | 7 | 0.27(0.15-0.43) | 0.78(0.40-0.97) |
| Hoxha et al.2012 ^12^ | 5 | 0 | 3 | 2 | 0.63(0.24-0.91) | 1.00(0.48-1.00 |
| Svobodova et al.2013 ^13^ | 8 | 2 | 29 | 1 | 0.22(0.10-0.38) | 0.33(0.01-0.91) |
| Svobodova et al.2012 ^20^ | 14 | 3 | 40 | 3 | 0.26(0.15-0.40) | 0.50(0.12-0.88) |
| Beck et al.2009 ^7^ | 6 | 0 | 5 | 3 | 0.55(0.23-0.83) | 1.00(0.29-1.00) |
| Ardalan et al.2013 ^11^ | 11 | 0 | 6 | 2 | 0.65(0.38-0.86) | 1.00(0.16-1.00) |
| Kanigicherla et al.2013 ^14^ | 12 | 1 | 38 | 18 | 0.24(0.13-0.38) | 0.95(0.74-1.00) |

Abbreviations: TP, true positive; FP, false positive; TN, true negative. FN, false negative; CI, confidence interval.

**Table S4.Raw data from serum anti-PLA2R in subgroups of treatment with or without immunosuppressor**

| **IS.Treatment** | **Number of patient** | | | | **Sensitivity (95%CI)** | **Specificity (95%CI)** |
| --- | --- | --- | --- | --- | --- | --- |
|  | **TP** | **FP** | **FN** | **TN** |  |  |
| **Yes** |  |  |  |  |  |  |
| Hoxha et al.2011 ^8^ | 28 | 0 | 24 | 5 | 0.54(0.39-0.68) | 1.00(0.48-1.00) |
| Qin et al.2011 ^9^ | 4 | 5 | 17 | 41 | 0.19(0.05-0.42) | 0.89(0.76-0.96) |
| Oh et al.2013 ^10^ | 3 | 2 | 16 | 7 | 0.16(0.03-0.40) | 0.78(0.40-0.97) |
| Beck et al.2009 ^7^ | 14 | 0 | 5 | 4 | 0.74(0.49-0.91) | 1.00(0.40-1.00) |
| Ardalan et al.2013 ^11^ | 17 | 0 | 6 | 2 | 0.74(0.52-0.90) | 1.00(0.16-1.00) |
| Svobodova et al.2012 ^20^ | 4 | 3 | 7 | 3 | 0.36(0.11-0.69) | 0.50(0.12-0.88) |
| Kanigicherla et al.2013 ^14^ | 42 | 1 | 48 | 18 | 0.47(0.36-0.57) | 0.95(0.74-1.00) |
| **No** |  |  |  |  |  |  |
| Hoxha et al.2011 ^8^ | 21 | 0 | 22 | 12 | 0.49(0.33-0.65) | 1.00(0.74-1.00) |
| Qin et al.2011 ^9^ | 49 | 5 | 11 | 41 | 0.82(0.70-0.90) | 0.89(0.76-0.96) |
| Oh et al.2013 ^10^ | 69 | 2 | 31 | 7 | 0.69(0.59-0.78) | 0.78(0.40-0.97) |
| Hoxha et al.2012 ^12^ | 60 | 0 | 13 | 15 | 0.82(0.71-0.90) | 1.00(0.78-1.00) |
| Svobodova et al.2013 ^13^ | 14 | 2 | 6 | 1 | 0.70(0.46-0.88) | 0.33(0.01-0.91) |
| Svobodova et al.2012 ^20^ | 16 | 3 | 6 | 3 | 0.73(0.50-0.89) | 0.50(0.12-0.88) |
| Beck et al.2009 ^7^ | 9 | 0 | 3 | 3 | 0.75(0.43-0.95) | 1.00(0.29-1.00) |

Abbreviations: IS, immunosuppressor; TP, true positive; FP, false positive; TN, true negative. FN, false negative; CI, confidence interval.

**Table S5 . Raw data from serum anti-PLA2R in subgroups of different sampling time from biopsy**

| **Sampling time from biopsy (mo)** | **Number of patient** | | | | **Sensitivity (95%CI)** | **Specificity (95%CI)** |
| --- | --- | --- | --- | --- | --- | --- |
|  | **TP** | **FP** | **FN** | **TN** |  |  |
| **0** |  |  |  |  |  |  |
| Qin et al.2011 ^9^ | 49 | 5 | 11 | 41 | 0.82(0.70-0.90) | 0.89(0.76-0.96) |
| Oh et al.2013 ^10^ | 69 | 2 | 31 | 7 | 0.69(0.59-0.78) | 0.78(0.40-0.97) |
| Hoxha et al.2012 ^12^ | 19 | 0 | 6 | 7 | 0.76(0.55-0.91) | 1.00(0.72-1.00) |
| Bajcsi et al.2013 ^19^ | 2 | 0 | 3 | 1 | 0.40(0.05-0.85) | 1.00(0.03-1.00) |
| Svobodova et al.2013 ^13^ | 14 | 1 | 6 | 1 | 0.70(0.46-0.88) | 0.50(0.01-0.99) |
| **﹥0** |  |  |  |  |  |  |
| Qin et al.2011 ^9^ | 4 | 5 | 17 | 41 | 0.19 (0.05-0.42) | 0.89(0.76-0.96) |
| Oh et al.2013 ^10^ | 3 | 2 | 16 | 7 | 0.16 (0.03-0.40) | 0.78(0.40-0.97) |
| Hoxha et al.2012 ^12^ | 41 | 0 | 7 | 8 | 0.85 (0.72-0.94) | 1.00(0.63-1.00) |
| Ardalan et al.2013 ^11^ | 17 | 0 | 6 | 2 | 0.74 (0.52-0.90) | 1.00(0.16-1.00) |
| Beck et al.2009 ^7^ | 22 | 0 | 6 | 6 | 0.79 (0.59-0.92) | 1.00(0.54-1.00) |
| Svobodova et al.2012 ^20^ | 12 | 1 | 33 | 0 | 0.27 (0.15-0.42) | 0.00(0.00-0.98) |
| Svobodova et al.2013 ^13^ | 24 | 3 | 51 | 3 | 0.32 (0.22-0.44) | 0.50(0.12-0.88) |
| Ardalan et al.2013 ^11^ | 17 | 0 | 6 | 2 | 0.74 (0.52-0.90) | 1.00(0.16-1.00) |
| Kanigicherla et al.2013 ^14^ | 42 | 1 | 48 | 18 | 0.47(0.36-0.57) | 0.95(0.74-1.00) |

Abbreviations: Mo, month; TP, true positive; FP, false positive; TN, true negative. FN, false negative; CI, confidence interval

**Table S6. Anti-PLA2R in idiopathic MN after remission**

| **Study (Author . Year)** |  | **Anti-PLA2R** | |
| --- | --- | --- | --- |
|  | **+** | **-** | [**Positive**](http://dict.youdao.com/search?q=positive&keyfrom=E2Ctranslation) [**incidence**](http://dict.youdao.com/search?q=incidence&keyfrom=E2Ctranslation) (%) |
| Qin et al.2011 ^9^ | 4 | 17 | 19.05 |
| Oh et al.2013 ^10^ | 3 | 16 | 15.79 |
| Svobodova et al.2013 ^13^ | 8 | 29 | 21.62 |
| Svobodova et al.2012 ^20^ | 14 | 40 | 25.93 |
| Kanigicherla et al.2013 ^14^ | 12 | 38 | 24.00 |
| Meneses et al.2014^23^ | 4 | 10 | 28.57 |
